# Enhancing immunity by engineering DAMPs

**DOI:** 10.18632/oncotarget.5251

**Published:** 2015-08-24

**Authors:** Felice Cervone, Frederick M. Ausubel, Giulia De Lorenzo

**Affiliations:** Department of Biology and Biotechnology “Charles Darwin,” Sapienza University of Rome, Rome, Italy

**Keywords:** Immunology and Microbiology Section, Immune response, Immunity, plant immunity, plant cell wall, pathogen resistance

Manipulation of innate immunity of living organisms to render them more rapid or sensitive in responding to microbial and environmental challenges is becoming a feasible strategy to combat diseases. Our knowledge of immunity-related molecules (signal molecules, receptors, interacting proteins, transducers, genes and defence proteins) is continually increasing in both animals and plants. In both kingdoms, recognition of invading microbes often activates powerful defences that restrict the growth of pathogens. Immunity is frequently triggered by microbe-associated molecular pattern molecules (MAMPs) [1]. However both plants and animals also recognize pathogens indirectly by the disruption of cellular homeostatic processes caused by infections. For example, in mammals, the presence of pathogens is sensed indirectly by the release of low molecular weight molecules such as oligonucleotides, uric acid, or ATP from damaged host tissues or alternatively, upon tissue injury, by the release of oligosaccharides, such as hyaluronan (HA) fragments from the extracellular matrix [2]. All these endogenous molecules act as “damage-associated molecular patterns” (DAMPs) that activate inflammatory responses and immunity. In particular, HA and derived fragments have been recently recognized to play an important role in the regulation of many immune-related diseases including cancer [3]. It is expected that strategies will be developed in the near future to control the expression of these molecules and their interactions to strengthen immune responses and improve health in both plants and animals.

**Figure 1:**
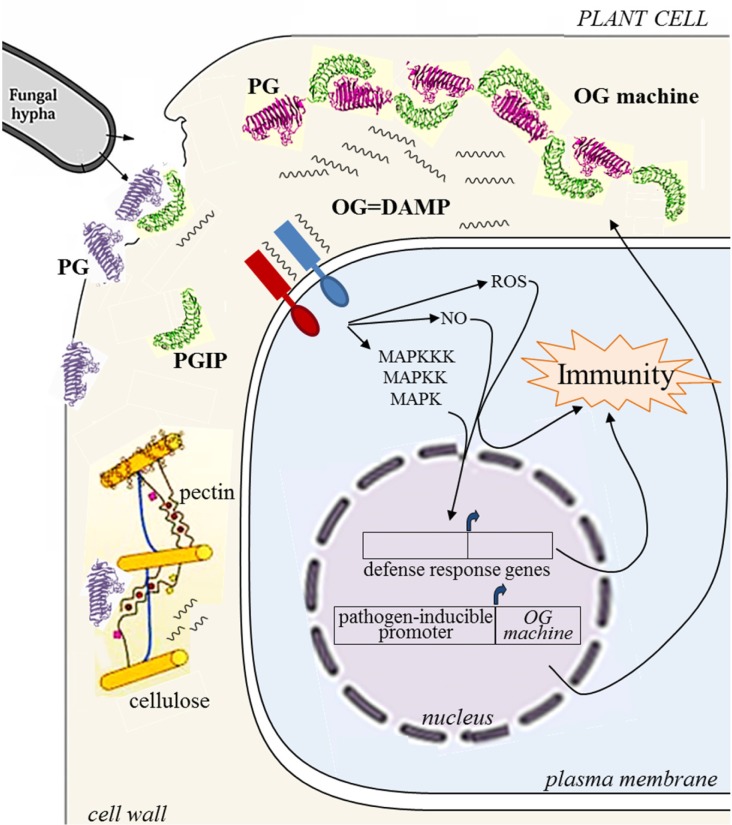
Schematic representation of the events occurring in the cell wall during a fungal attack of a plant tissue Polygalacturonase (PG) secreted by the pathogen breaches the wall by degrading the pectin component. In the presence of the inhibitor PGIP degradation is slowed down and the formation of signal molecules (OG) for the activation of immunity is favoured. Expression under a pathogen-inducible promoter of a PG-PGIP fusion protein named OG-machine enhances the accumulation of OG and immunity.

In a recent paper [4], we have investigated and verified the possibility of engineering in plants the controlled release of DAMPs derived from the extracellular matrix (the plant cell wall), to enhance immune responses and confer resistance against several microbial pathogens. The concept of engineering DAMPs in plants is quite new and may potentially be applied to the animal field. We have exploited the knowledge that oligogalacturonides (OG) with a degree of polymerization between 10 and 15, i.e. oligosaccharides released by the action of microbial polygalacturonases from homogalacturonan, a component of the plant cell wall pectin, are capable of activating a wide range of defence responses. In other words, plants are capable of sensing a breach in the wall through the perception of oligogalacturonides and promptly defend themselves. Activation of immunity mediated by oligogalacturonides occurs in many plants and is effective against many microbes. The cascade linking the perception of oligogalacturonides to the activation of immunity has been partly elucidated [5, 6]. During microbial infections, the generation of elicitor-active oligogalacturonides is promoted by plant-encoded polygalacturonase inhibitor proteins (PGIP), which block the complete hydrolysis of homogalacturonan and favour the accumulation of elicitor-active oligomers vs. the formation of elicitor-inactive oligomers and monomers of galacturonic acid [6, 7]. We devised an experimental strategy to test whether transgenic plants engineered to accumulate equimolar levels of polygalacturonase and PGIP generate elicitor-active oligogalacturonides. We constructed a chimeric protein by fusing a polygalacturonase from a fungal pathogen to a plant-derived PGIP and showed that transgenic *Arabidopsis thaliana* plants expressing the enzyme-inhibitor chimera indeed accumulate elicitor-active oligogalacturonides. The chimera was named the “OG-machine”. Expression of the OG-machine under the control of a pathogen-induced promoter confers increased resistance to Arabidopsis against a variety of pathogens, pointing to a possible biotechnological use of the OG-machine to protect crop plants against a broad range of microbial diseases (Figure 1). Importantly, because the OG-machine confers resistance against both fungi and bacteria with different lifestyles, it may be particularly useful against field and postharvest pathogens and saprophytes that cause important crop losses and mycotoxin contamination.

The disease resistance genes most widely used in traditional and genetic engineering breeding programs encode receptor proteins that trigger an immune response upon recognition of specific MAMPs or specific pathogen genotypes. However, resistance conferred by these proteins is restricted and typically lacks durability because pathogens continually evolve genotypes that are able to evade recognition [8]. Engineering the endogenous production of DAMPs may circumvent this problem and, at the same time, confer to a crop a broad-spectrum of resistance against many microbes.

Our work provides direct evidence of *in vivo* activation of the innate immune responses upon release of DAMPs and also provides a tool to investigate some important aspects of plant biology. It is known, for example, that trade-off occurs between the growth potential and the defence capacity of plants. Maintenance of immunity is costly and immune responses are typically counterbalanced by decreasing the allocation of resources to biomass production. Plants that constitutively express defence responses are often dwarf or die. Like in animals, it appears that an exaggerated release of endogenous danger signals leads to a deleterious hyperimmune response. It is noteworthy that when the OG-machine is induced at high levels, plant growth is significantly reduced or arrested, indicating that high concentrations of endogenous oligogalacturonides interfere with normal developmental programs. However, by using an appropriate promoter (*PR-1*: *PATHOGENESIS RELATED GENE 1*), which senses the presence of pathogens and locally activates transcription, we were able to engineer the release of oligogalacturonides in a controlled way avoiding undesiderable changes in growth and development. Many other pathogen-inducible promoters are available in plants for the purpose described in this editorial.
